# Consumption of carbapenems in different intensive care units in a Saudi tertiary care hospital

**DOI:** 10.1186/2047-2994-4-S1-P181

**Published:** 2015-06-16

**Authors:** HH Balkhy, A Al Othman, IH Baffoe Bonnie, YM Arabi, A El-Saed

**Affiliations:** 1 Infection control, King Abdulaziz Medical City, Riyadh, Saudi Arabia; 2 Department of Medicine, King Abdulaziz Medical City, Riyadh, Saudi Arabia; 3 Intensive Care Department, King Abdulaziz Medical City, Riyadh, Saudi Arabia

## Introduction

Surveillance of antimicrobial consumption is a necessary step to develop and monitor policies to limit antimicrobial overuse/misuse and to decrease the risk of bacterial resistance. In Saudi Arabia, such surveillance data are lacking.

## Objectives

To quantify the carbapenems use in intensive care unit (ICU) setting and to detect any variability by ICU type.

## Methods

We conducted a prospective surveillance study in eight different ICUs; five adult and three pediatric/neonatal ICUs at King Abdulaziz Medical City, Riyadh, Saudi Arabia, Riyadh, from October 2012 through December 2013. We estimated the consumption of carbapenems as days of use (which was defined as the aggregate sum of days for which any amount of meropenem or imipenem were administered) per 1000 bed-days or 100 admissions.

## Results

We recorded carbapenem use for more than 3500 bed-days during 15 months in 8 ICUs having a total 131 bed capacity. The overall carbapenem use was estimated at 133.7 days of use per 1000 bed-days and 150.8 days of use per 100 admissions. The use per bed-days was highest in adult medical surgical ICU (357.0) followed by adult step-down (232.4) and burn (195.4) ICUs but lowest in neonatal ICU (27.7) and adult cardiothoracic ICU (64.9). The use per admissions was highest in adult step-down ICU (467.8) followed by burn (394.9) and adult medical surgical (384.0) ICUs but lowest in adult cardiothoracic (31.2) and pediatric medical-surgical (58.9) ICUs.

## Conclusion

Approximately, our ICU patients use carbapenems one-eighth of the days spent. There is wide variability in carbapenem use in different ICUs that warrant ICU-specific reporting and pinpoint areas that need more management attention. There is need to identify patient and physician characteristics associated with this high carbapenem consumption.

## Disclosure of interest

None declared.

